# Non-response bias in the analysis of the association between mental health and the urban environment: a cross-sectional study in Brussels, Belgium

**DOI:** 10.1186/s13690-023-01118-y

**Published:** 2023-07-07

**Authors:** Madeleine Guyot, Ingrid Pelgrims, Raf Aerts, Hans Keune, Roy Remmen, Eva M. De Clercq, Isabelle Thomas, Sophie O. Vanwambeke

**Affiliations:** 1 Earth & Life Institute, Université catholique de Louvain, Louvain-La-Neuve, Belgium; 2 Louvain Institute of Data Analysis and Modeling in Economics and Statistics, Université catholique de Louvain, Louvain-La-Neuve, Belgium; 3 Chemical and Physical Health Risks, Sciensano (Belgian Institute of Health), Brussels, Belgium; 4 Epidemiology and Public Health, Sciensano (Belgian Institute of Health), Brussels, Belgium; 5 Applied Mathematics, Computer Science and Statistics, Ghent University, Ghent, Belgium; 6 Division Ecology, Evolution and Biodiversity Conservation, KU Leuven, Louvain, Belgium; 7 Faculty of Medicine and Health Sciences Department of Primary and Interdisciplinary Care, University of Antwerp, Wilrijk, Belgium

**Keywords:** Mental Health, Non-response, Urban Environment, Brussels

## Abstract

**Background:**

This paper aims at analysing the impact of partial non-response in the association between urban environment and mental health in Brussels. The potential threats of the partial non-response are biases in survey estimates and statistics. The effect of non-response on statistical associations is often overlooked and evidence in the research literature is lacking.

**Methods:**

Data from the Belgian Health Interview Survey 2008 and 2013 were used. The association between non-response and potential determinants was explored through logistic regressions.

**Results:**

Participants with low income, low educational levels, lower or higher age or in households with children were less likely to respond. When adjusting for socio-economic variables, non-response was higher in areas which are less vegetated, more polluted or more urbanised. Because the determinants of non-response and depressive disorders were similar, it is reasonable to assume that there will be more people with mental health problems among the non-respondents. And because more non-responses were found in low vegetation areas, the protective association between green spaces and mental health may be underestimated.

**Conclusion:**

Our capacity to measure the association between the urban environment and health is affected by non-response in surveys. The non-random spatial and socio-economic distribution of this bias affects the research findings.

**Supplementary Information:**

The online version contains supplementary material available at 10.1186/s13690-023-01118-y.

## Background

The issue of non-response and incomplete data, that often confronts population studies such as health surveys, is a well-known challenge in epidemiology [1, 2] that has already been extensively studied [3–9]. Non-response can occur at different stages of the survey and varies from the refusal to participate (initial non-response) to a non-response to one of the questions (partial non-response). While the determinants of initial non-response are difficult to study (via auxiliary data as in Ekholm et al*.*, 2010 or via non-responder questionnaire as in Jaehn et al*.*, 2020), partial non-response has the advantage that it can be studied on the basis of participants' partial responses [3, 4, 9].

Personal characteristics, such as economic status, are important in the variation of non-response [5–8]. In the case of the Belgian Health Interview Survey (BHIS), determinants of partial non-response have already been evaluated by Berete et al. [3]: non-response is more frequent among youngsters, non-Belgians, low educational levels, lower income, residents of Brussels and Wallonia, and people with poor perceived health. Partial non-response is also strongly associated with the interviewer: it explains almost half (46.6%) of the variability in partial non-response. Other studies [4, 9] on partial non-response have additionally shown that non-response is greater for men and for unskilled workers. The potential threats of this partial non-response are biases in survey estimates and statistics. The effect of non-response on statistical associations is often overlooked and evidence in the research literature is lacking [2].

Understanding the association between the urban environment and mental health is a challenging exercise. Cities are complex systems, with urban mental health outcomes affected by many interactions [10]. This topic has been attracting a lot of interest and the literature has grown substantially over the past few years [11–18]. A study conducted in Brussels concluded that exposure to traffic-related air pollution (black carbon, NO_2_, PM_10_) was positively associated with higher odds of depressive disorders but no association between mental health and green surroundings, noise, or building morphology could be demonstrated [19]. Concerning vegetation, these results differ from many studies that describe how higher levels of exposure to greenness can reduce levels of depressive symptoms [20]. This raises the question of why there is no association in the case of Brussels.

Personal characteristics, such as economic status, are also important in mental health while the urban environment appears to be a less important determinant [21]. Moreover, in Brussels, as in other cities, socio-spatial inequalities complicate matters: environmental factors are strongly correlated with socio-economic ones [22]. It is therefore difficult to disentangle the variance due to socio-economic status from the variance due to the urban environment.

If personal characteristics play an important role in explaining non-response and mental health status, we can question the magnitude of bias generated by non-response in the analysis of the association between urban environment and mental health. This paper therefore aims at analysing, characterising and discussing the impacts of non-responses in this association with the example of Brussels.

## Methods

### Study area

Brussels is the capital of Belgium, centrally located in the country. The city extends into three administrative regions: the Brussels-Capital Region (BCR), Flanders and Wallonia [23].We focus here on the urban area encompassed within the administrative border of the Brussels-Capital Region (BCR) because the Region was more intensively sampled compared to the rest of Belgium and because of data consistency issues for the urban environment indicators. The BCR is divided into 19 municipalities. It covers 161.38 km^2^ and has 1.2 million inhabitants (source: Statistics Belgium – 01/01/2018), which means an average density of 7438 inhabitants per square kilometre.

### Study design

The study presented here consists of a secondary analysis of a population-based, cross-sectional study of the association between urban environment and mental health in Brussels, Belgium. Results from the original research could not demonstrate any association between urban environment (green surroundings, noise, building morphology) and mental health except for traffic-related air pollution (black carbon, NO_2_, PM_10_) exposure which was positively associated with higher odds of depressive disorders [19].

### Study population and data

The Belgian Health Interview Surveys (BHIS) of 2008 and 2013 were used. We kept participants aged over 15 (minimum age to fill the self-administered questionnaire) and living at the place of residence for at least one year (*n* = 4,355).

In the BHIS, three types of non-response can be highlighted: (i) Initial non-response: refusal to participate in the survey [24], not addressed in this paper; (ii) Self-administered questionnaire (SAQ) non-response: for those over 15 years of age, the survey consists in two parts: a face-to-face (F2F) interview and a SAQ, the latter of which is not filled out; and (iii) Item non-response: at various points of the survey some questions were not answered by the participants. These last two types can be designated as partial non-response. Questions related to mental health are particularly sensitive. They are generally not asked in a F2F interview to avoid desirability bias and are included in a SAQ, as is the case for the BHIS.

### Mental health and non-response definition

The response to six indicators related to mental health was extracted from the BHIS [25]: *Energy level (SF-36 ‘vitality scale’)* [based on 4 items in SAQ] [26], *General Health Questionnaire (GHQ-12)* [based on 13 items in SAQ] [27], *Self-reported depression* during the last year [based on 1 item in F2F interview], *Depressive disorder* [based on 13 items in SAQ], *Anxiety disorder* [based on 10 items in SAQ] and *Sleeping disorder* [based on 3 items in SAQ] (SCL-90-R subscales) [28].

Non-response to a mental health indicator means that there are not enough items answered to compute the indicator. The reason may be either that the SAQ was not completed or that some or all of the items related to an indicator were not answered. If at least 75% of the items related to an indicator have valid responses, the missing item(s) are substituted by the mean value of the valid responses. For example, if 1 to 3 items out of the 12 items of the GHQ scale have missing values, the GHQ is still computed; whereas if more than 3 items are missing, the indicator score is considered missing [29].

### Urban environment

The urban environment was measured by eight indicators computed at the residence address of the BHIS participants. Urban greenness was assessed at three different geographical levels (residence, street, neighbourhood). At residence level, v*iew of green* was determined by the ratio of the total green area from Google Street View panorama (~ picture 360°) to the total area of the panorama. At street level, *Linear tree density* was defined as the ratio between the number of trees (point data from Urbis) [30] and the street length and *Street visible vegetation coverage* is the vegetation coverage (from Brussels Environment) [31] on the street, and 10 m on either side. At neighbourhood level, *Vegetation coverage* was defined as the ratio of vegetation coverage (from Brussels Environment) in a 1000 m circle around the respondent’s residence. The built-up environment was assessed by two indicators at the street level: the *Street canyon effect* which is the ratio between average building height and average open space width and the *Street corridor effect* which is the ratio between parallel facades length and street length. *Noise* was extracted from noise from multiple sources map (day – evening – night noise level, Lden) from Bruxelles Environnement for the years 2006 and 2011 at residence level. *Black carbon* exposition was defined as the annual average in the year of BHIS participation at residence level [32–34]. See Pelgrims et al*.* [19] for more detailed descriptions of the indicators. These indicators were missing when the health survey could not be coupled with the participant’s address (in Brussels). To address this, a ‘no data’ category was added to each indicator with missing value.

### Socio-economic status

Five socio-economic indicators were used: *Reported household income*, *Age*, *Gender*, *Family composition* and *Highest educational level in the household*. These indicators were extracted from the BHIS. Non-responses were observed if the item has not been answered. To address this, a ‘no answer’ category was added to each indicator with non-responses.

### Statistical analyses

The frequency of non-response was summarised for each mental health and socio-economic indicator. Descriptive statistics were calculated to account for the sampling strategy. ‘The weight for each sampled individual in the BHIS is the product of the reciprocal of the selection probability within a household and of a post stratification factor for each province according to age, gender, household size and quarter of the year in which the interview was done’ (Scientific Institute of Public Health, 2013).

In order to assess the association of socio-economic and urban environmental with non-response, logistic regressions with the non-response as dependent variable were computed: no missing data (0) vs. at least one non-response among six mental health indicators (1). Taking non-response to at least one mental health outcome as a proxy for non-response is a somewhat crude generalisation. However, it reflects a practice: if we want to analyse a complete case, i.e. take only those individuals who responded to all the questions we are interested in, we will then remove all individuals who did not respond to at least one question. Our purpose here was first to identify the characteristics of those excluded from the complete case by running models for socio-economic indicators. Secondly, we aimed at analysing whether these individuals were concentrated in specific environments by running single-exposure models for urban environment indicators (models A) and adjusted models (with gender, age, reported household income and year of the BHIS) for urban environment indicators (models B).

In order to assess the differences and similarities between the socio-economic characteristics of non-respondents and participants with depressive disorders, two groups of logistic regressions were performed for socio-economic variables: (i) with depressive disorders as dependent variable (models C) and (ii) with the non-response to depressive disorders related questions as dependent variable (models D).

Correct estimates and valid inferences of odds ratio (OR) were obtained by taking into account the survey weights, strata and clusters relative to the sample design. All analyses were performed using the statistical software R [35] using the survey package [36].

## Results

### Data description

A total of 4355 residents of the BCR were included in the study population. Table 1 describes all considered variables.

**Table 1:** Characteristics of the study population (weighted percentages and weighted mean)

Socio-economic status	
**Reported household income** %/N	
Quartile 1 (low)	20.66%/893
Quartile 2	18.53%/866
Quartile 3	21.64%/926
Quartile 4 (high)	22.24%/904
No answer	16.93%/766
**Reported household income [€]** mean [IQR]	1525.03 [1473.46–1576.61]
**Age** %/N	
15–24	13.81%/536
25–44	37.39%/1439
45–64	30.06%/1317
65 +	18.74%/1063
**Age** median [IQR]	46.08 [45.35–46.81]
**Gender** %/N	
M	48.19%/1986
F	51.81%/2369
**Year of the BHIS** %/N	
2008	45.26%/2203
2013	54.74%/2152
**Family composition** %/N	
Single	28.79%/1176
Couple with child (ren)	33.81%/1469
Couple without child (ren)	17.20%/809
One parent with child (ren)	11.25%/502
Other/unknown	8.95%/399
**Highest educational level in the household** %/N	
Higher	45.49%/1882
Higher secondary	26.13%/1142
Lower secondary	13.68%/631
No diploma or primary education	11.87%/556
No answer	2.84%/144
**Environmental factors**	
**Street canyon effect** mean [IQR]	0.67 [0.65–0.69]
**Street corridor effect [0–2]** mean [IQR]	1.66 [1.64–1.69]
**Street corridor effect** %/N	
< median	23.87%/668
> = median	24.90%/652
maximum	50.14%/1209
No data	1.09%/33
**Linear tree density** mean [IQR]	0.09 [0.08–0.09]
**Linear tree density** %/N	
No tree	27.21%/669
< median	35.62%/932
> = median	36.08%/928
No data	1.09%/33
**Vegetation coverage (1 km buffer)** mean [IQR]	0.38 [0.37–0.39]
**Street visible vegetation coverage (within 10 m)** mean [IQR]	0.17 [0.16–0.19]
**View of green** mean [IQR]	14.52 [13.98–15.07]
**Noise from multiple sources Lden [dB]** mean [IQR]	51.29 [51.04–51.55]
**Black carbon** mean [IQR]	2.30 [2.27–2.33]

Overview of non-responses among the study population is displayed in Table 2. The number of individuals refers to the number of respondents in the sample while the percentage is weighted to represent the study population. In our sample of 4,355 individuals, the SAQ was not available for 1,602 participants (35.48% of the population). For 18.97%, SAQ was required but not available and for 16.51%, SAQ was not required and not available (when the interview is done by a proxy such as a parent). Non-response to mental health items (excluding SAQ non-response) ranged from 1.03% (*Self-reported depression*) to 7.33% (*Energy level*). In the subset of participants for whom the SAQ was available and who answered all mental health and socio-economic items, data was available for 1,929 individuals (45.92% of the initial population).

**Table 2:** Non-response in the study population. No answer (NA) is due to an item non-response or to the self-administered questionnaire (SAQ) not filled out (rows in grey). Complete cases are individuals with all socio-economic (SE) and/or mental health (MH) indicators answered. Weighted percentages

	**Initial sample**^**a**^		**SAQ subset°**	
	**Obs**	**%**	**Obs**	**%**
Initial sample	4355	100.00%		
NA Reported household income (SE)	766	16.93%		
NA Age (SE)	0	0.00%		
NA Gender (SE)	0	0.00%		
NA Family composition (SE)	399	8.95%		
NA Highest educational level in the household (SE)	144	2.84%		
NA Self-reported depression (MH)	44	1.03%		
SAQ required but not available	842	18.97%		
SAQ not required and not available	760	16.51%		
SAQ not required but available	110	2.50%		
SAQ required and available	2643	62.02%		
SAQ available		64.52%	2753	100.00%
NA General Health Questionnaire (GHQ-12) (MH)	1723	38.17%	121	2.70%
NA Energy level (SF-36 ‘vitality scale’) (MH)	1918	42.80%	316	7.33%
NA Depressive disorder (MH)	1793	39.51%	191	4.04%
NA Anxiety disorder (MH)	1801	39.75%	199	4.27%
NA Sleeping disorder (MH)	1811	39.91%	209	4.44%
Complete case for mental health	2285	53.93%		
Complete case for mental health and socio-economic	1929	45.92%		

^a^ whole study population; ° participants with SAQ available

### Determinants of partial non-response

**Fig. 1:**
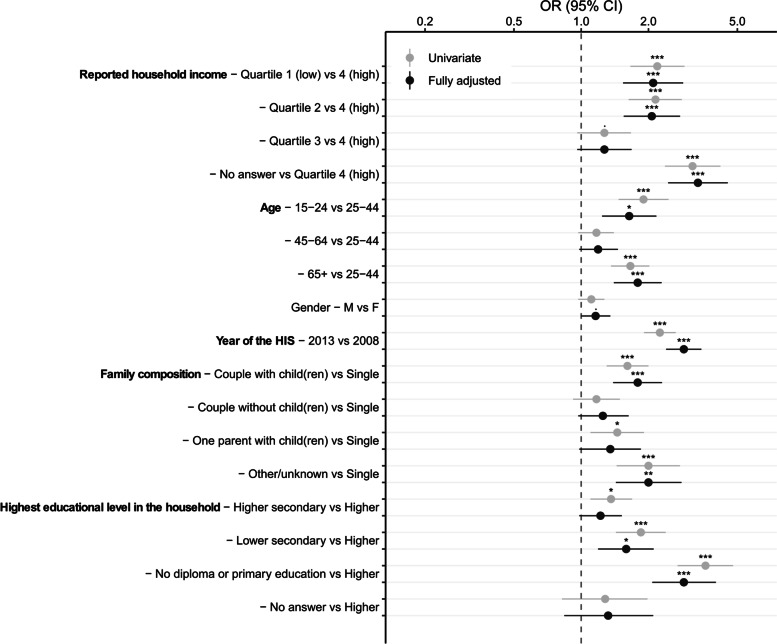
Association between non-response and socio-economic indicators (univariate regression models and fully adjusted regression model). Levels of significance: ‘***’ = *p* < 0.0001, ‘**’ = *p* < 0.001, ‘*’ = *p* < 0.01, ‘·’ = *p* < 0.05

Figure 1 shows the association between socio-economic variables and non-responses through univariate models and fully adjusted model. These models are also displayed in Additional File 1. Considering that the coefficients between the univariate models and the fully adjusted model are very similar, only the coefficients of the univariate models will be discussed below. Participants in the lower socio-economic quartiles (Quartile 1 and Quartile 2) were less likely to respond than those in the higher quartile (Quartile 4) (*p* < 0.001, OR = 2.19, 95% CI = 1.66–2.90 and *p* < 0.001, OR = 2.15, 95% CI = 1.63–2.82 respectively). Compared to 25–44 yr, older (65 + yr) and younger (15–24 yr) participants were less likely to respond (*p *< 0.001, OR = 1.66, 95% CI = 1.36–2.02 and *p* < 0.001, OR = 1.9, 95% CI = 1.47–2.46 respectively). Compared to single participants, couples with child (ren) and one parent with child (ren) were less likely to respond (*p* < 0.001, OR = 1.61, 95% CI = 1.3–2 and *p* = 0.008, OR = 1.45, 95%CI = 1.1–1.91 respectively). Finally, participants from households with a lower educational level (higher secondary, lower secondary and no diploma or primary education) were less likely to respond than those with a higher education (after secondary) and the effect size increases as the level of education decrease (*p* = 0,004, OR = 1.36, 95% CI = 1.1–1.69 and *p* < 0.001, OR = 1.85, 95% CI = 1.43–2.39 and *p* < 0,001, OR = 3.6, 95% CI = 2.7–4.79 respectively).

**Fig. 2:**
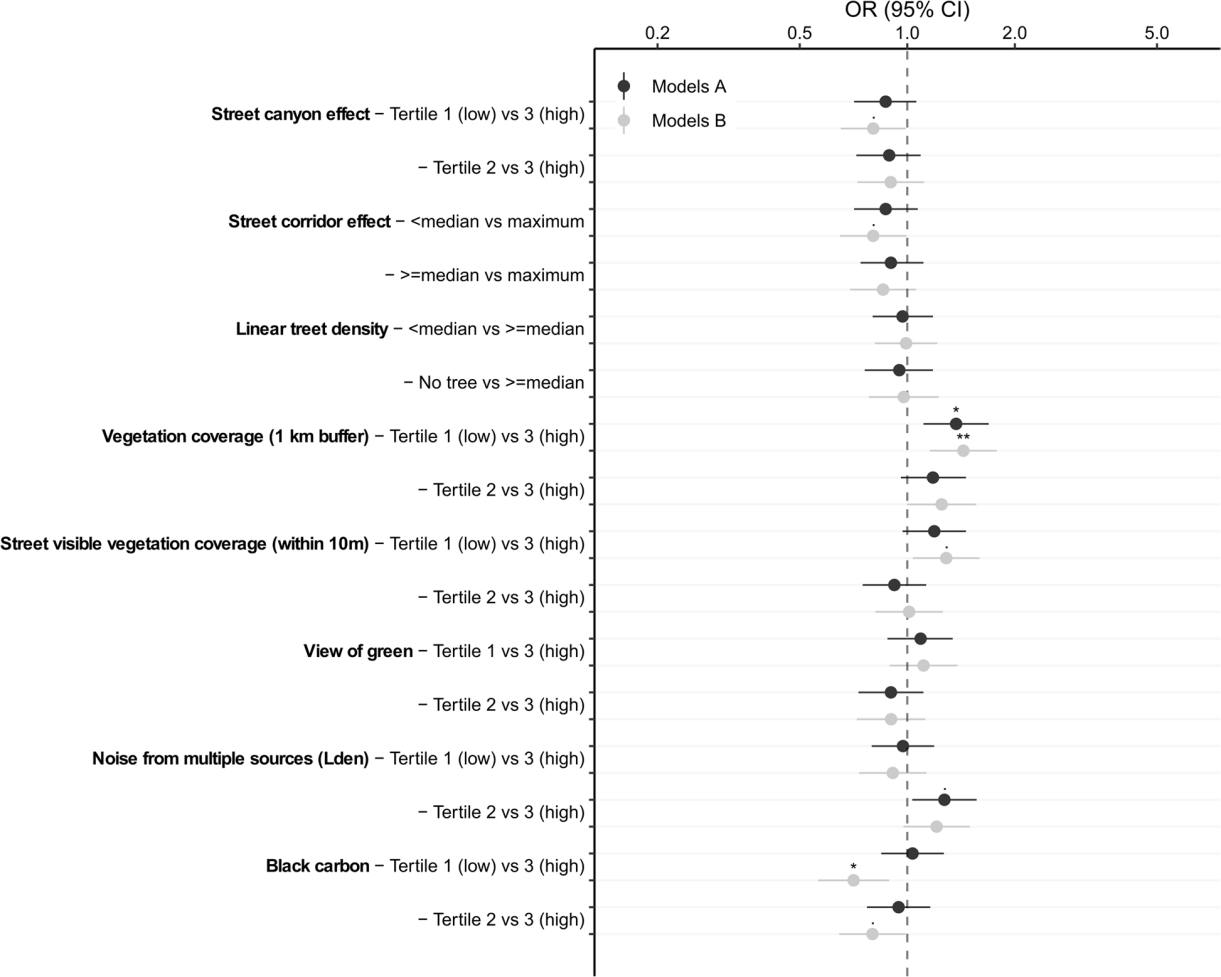
Association between non-response and urban environment indicators. Models A are univariate regression models. Models B are adjusted regression models for gender, age, reported household income and year of the BHIS. Levels of significance: ‘***’ = *p* < 0.0001, ‘**’ = *p* < 0.001, ‘*’ = *p* < 0.01, ‘·’ = *p* < 0.05

Figure 2 shows the association between urban environment indicators and non-response through univariate models (Models A) and adjusted models (Models B) for gender, age, reported household income and year of the BHIS. These models are also displayed in Additional File 2. When adjusted for socio-economic variables, non-response was higher in areas with lower amounts of vegetation cover (vegetation at 1 km buffer, *p* = 0.001, OR = 1.44, 95% CI = 1.16–1.78 and street visible vegetation, *p* = 0.022, OR = 1.29, 95% CI = 1.04–1.59), lower in less polluted areas (black carbon, *p* = 0.003, OR = 0.71, 95% CI = 0.56–0.89) and in less urbanised areas (street canyon, *p* = 0.04, OR = 0.8, 95% CI = 0.65–0.99 and street corridor effect, *p* = 0.044, OR = 0.8, 95% CI = 0.65–0.99).

### Comparison of partial non-response determinant and depressive disorders determinants

**Fig. 3:**
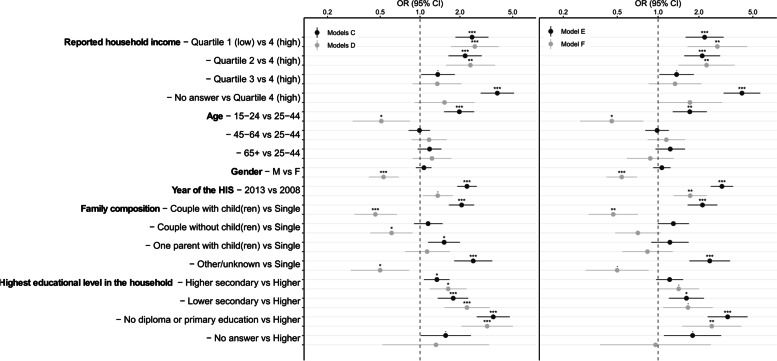
Association between non-response to depressive disorders related questions and socio-economic indicators (univariate regression models C and fully adjusted regression model E) and between depressive disorders and socio-economic indicators (univariate regression models D and fully adjusted regression model F). Levels of significance: ‘***’ = *p* < 0.0001, ‘**’ = *p* < 0.001, ‘*’ = *p* < 0.01, ‘·’ = *p* < 0.05

Figure 3 shows the associations between socio-economic variables and non-response to depressive disorders related questions (univariate regression models C and fully adjusted regression model E) and the associations between socio-economic variables and depressive disorders (univariate regression models D and fully adjusted regression model F). These models are also displayed in Additional File 3 and 4. It should be noted that the two populations studied are different: population of models D and F is limited to the respondents to depressive disorders related questions only. The comparison of the two types of models nevertheless allows us to have an overview of the similarities and differences even if it cannot be compared on a more formal basis. The determinants of non-response are quite similar to the determinants of depressive disorders. Odds ratios are similar for household income, age (except for 15–24 y) and education level, while there are substantial differences for family composition and gender.

## Discussion

Our results confirm that personal characteristics play a major role in explaining both non-response and mental health status. The socio-economic variables associated with non-response found in this paper (Fig. 1) are similar to those found in the literature: non-response is more frequent among young [3, 6], low educated [3, 4, 7, 9] and low income [3] inhabitants. Family composition or type of household is not a common studied factor in the literature; Ekholm et al*.* [6] and Jaehn et al*.* [7] found that married people are more likely to respond to the survey than unmarried people but it is difficult to link this finding to our results because marital status has not been taken into account as such and being in a couple is mixed with having children. A higher non-response rate for men than for women is often found in the literature [4, 6, 9] but was not found in our study.

Based on the comparison between the personal characteristics associated with non-response and depressive disorders in the results (Fig. 3), it is reasonable to assume that there will be more people with mental health problems within the group of non-respondents compared to the group of respondents. This is coherent with the literature that examined poor subjective health [7, 9], chronic condition [3] or long-term health condition [4] as a determinant for non-response.

With these results, we can extrapolate the effect of non-response to the association between mental health and the urban environment. Considering the association between vegetation and mental health, several studies on the topic have shown that vegetation is a protective factor for mental health [37, 38]. However, in a previous study using the same data as this paper [19], no association was found. With our results, it can be assumed that there would be proportionally higher number of respondents with mental disorders and low vegetation cover (${A}_{true}$) than actually observed due to non-responses $({A}_{observed})$ because both low vegetation cover and mental health problems are associated with a higher probability for non-responses. Indeed, results show that the personal characteristics associated with mental health have strong similarities with those of non-response and significantly more non-responses are found in places with lower vegetation cover.

Considering the equation of an odds ratio ($OR$) with $A$ as the number of respondents with mental disorder (MD +) and exposed to low green space cover (LGS +), $B$ as the number of respondents with no mental disorder (MD-) and exposed to low vegetation cover (LGS +), $C$ as the number of respondents with mental disorder (MD +) and high vegetation cover (LGS-) and $D$ as the number of respondents with no mental disorder (MD-) and high vegetation cover (LGS-) (Table 3):

**Table 3:** Contingency table of disease vs. exposure to calculate the odds ratio

		**Disease or condition**	
		Mental disorder (MD +)	No mental disorder (MD-)
**Exposure of factor**	Low green space cover (LGS +) = exposed	A	B
	High green space cover (LGS-) = control	C	D

$$OR= \frac{A* D}{B* C}$$

When this OR is greater than 1, it indicates that the odds of exposure (to low green space) among case-patients (mental disorder, MD +) are greater than the odds of exposure among controls. The exposure (to low green space cover) is interpreted as a risk factor for the disease or condition (mental disorder).

If *A*_*true*_ > *A*_*observed*_ we may conclude that *OR*_*true*_ > *OR*_*observed*_. Therefore, the risk of having a mental disorder associated with exposure to low green space cover is underestimated (*OR*_*observed*_ < *OR*_*true*_), i.e. the odds ratio observed is smaller than the true odds ratio.

Despite the strength of the arguments presented and the valuable insights gained from this study, we have to acknowledge missing data cannot be recovered. Without complete data on all individuals, it is challenging to ascertain associations and their implications accurately. Future prospective studies can provide a more comprehensive understanding of the relationship between personal characteristics, non-response, mental health, and the urban environment.

## Conclusions

In conclusion, we here show that the risk of low green space cover (or, conversely, the protective effect of high green space cover) is underestimated using the BHIS data. Indeed, the capacity to measure the association between urban environment and health is affected by non-response in surveys. The non-random spatial and socio-economic distribution of this bias affects the parameter estimates in the statistical models. In a sense, non-responses may be seen as a cause of a loss of statistical power, leading to a lower probability to statistically detect differences if such differences would exist. This may explain why when studying environment and human health, results are sometimes inconclusive or inconsistent with the literature. The environment and human health association is particularly challenging to study as we are dealing with multifactorial elements. More attention should be given to understand the biases that can be encountered due to non-responses and to the non-random character of this bias. A combination of methods or a mixed-method approach (quantitative and qualitative) can help to obtain a comprehensive understanding of these highly complex topics.

## Supplementary Information

**Additional file 1. ** Association between non-response and socio-economic indicators.

**Additional file 2. ** Association between non-response and urban environment indicators. Models A are univariate regression models. Models B are adjusted regression models for gender, age, reported household income and year of the BHIS.

**Additional file 3. ** Association between non-response to depressive disorders related questions and socio-economic indicators (univariate regression models C) and between depressive disorders and socio-economic indicators (univariate regression models D).

**Additional file 4. ** Association between non-response to depressive disorders related questions and socio-economic indicators (fully adjusted regression model E) and between depressive disorders and socio-economic indicators (fully adjusted regression model F).

## Data Availability

The datasets generated and analysed during the current study are not publicly available due to privacy.
