# Supplementary material for: Non-response bias in the analysis of the association between mental health and the urban environment: a cross-sectional study in Brussels, Belgium
Source: Arch Public Health. 2023 Jul 7;81:129. doi: 10.1186/s13690-023-01118-y (PMC10327324; doi:10.1186/s13690-023-01118-y)
Supplement: Supplementary file 1 — Additional file 1. Association between non-response and socio-economic indicators. [file 13690_2023_1118_MOESM1_ESM.docx]

**Additional File 1.** Association between non-response and socio-economic indicators.

|  |  | **Univariate models** |  | **Fully adjusted model** |  |
| --- | --- | --- | --- | --- | --- |
|  |  | OR (95% IC) | p value | OR (95% IC) | p value |
| **Reported household income** | Quartile 1 (low) vs 4 (high) | 2.19 (1.66-2.9) | 0.000 | 2.1 (1.54-2.86) | 0.000 |
|  | Quartile 2 vs 4 (high) | 2.15 (1.63-2.82) | 0.000 | 2.07 (1.55-2.77) | 0.000 |
|  | Quartile 3 vs 4 (high) | 1.27 (0.96-1.67) | 0.096 | 1.27 (0.96-1.68) | 0.094 |
|  | No answer vs Quartile 4 (high) | 3.15 (2.37-4.2) | 0.000 | 3.33 (2.45-4.53) | 0.000 |
| **Age** | 15–24vs 25–44 | 1.9 (1.47-2.46) | 0.000 | 1.64 (1.24-2.17) | 0.001 |
|  | 45–64vs 25–44 | 1.17 (0.97-1.4) | 0.104 | 1.19 (0.98-1.46) | 0.082 |
|  | 65+ vs 25–44 | 1.66 (1.36-2.02) | 0.000 | 1.79 (1.4-2.29) | 0.000 |
| **Gender** | M vs F | 1.11 (0.97-1.27) | 0.125 | 1.16 (1-1.35) | 0.047 |
| **Year of the BHIS** | 2013 vs 2008 | 2.25 (1.91-2.65) | 0.000 | 2.88 (2.4-3.45) | 0.000 |
| **Family composition** | Couple with child (ren) vs Single | 1.61 (1.3-2) | 0.000 | 1.79 (1.39-2.3) | 0.000 |
|  | Couple without child (ren) vs Single | 1.17 (0.92-1.49) | 0.197 | 1.25 (0.97-1.63) | 0.090 |
|  | One parent with child (ren) vs Single | 1.45 (1.1-1.91) | 0.008 | 1.35 (0.98-1.85) | 0.068 |
|  | Other/unknown vs Single | 2 (1.44-2.77) | 0.000 | 2 (1.43-2.81) | 0.000 |
| **Highest educational level in the household** | Higher secondary vs Higher | 1.36 (1.1-1.69) | 0.004 | 1.22 (0.98-1.52) | 0.073 |
|  | Lower secondary vs Higher | 1.85 (1.43-2.39) | 0.000 | 1.59 (1.19-2.11) | 0.002 |
|  | No diploma or primary education vs Higher | 3.6 (2.7-4.79) | 0.000 | 2.88 (2.08-4.01) | 0.000 |
|  | No answer vs Higher | 1.28 (0.82-1.98) | 0.276 | 1.32 (0.84-2.1) | 0.231 |
