# Supplementary material for: Non-response bias in the analysis of the association between mental health and the urban environment: a cross-sectional study in Brussels, Belgium
Source: Arch Public Health. 2023 Jul 7;81:129. doi: 10.1186/s13690-023-01118-y (PMC10327324; doi:10.1186/s13690-023-01118-y)
Supplement: Supplementary file 2 — Additional file 2. Association between non-response and urban environment indicators. Models A are univariate regression models. Models B are adjusted regression models for gender, age, reported household income and year of the BHIS. [file 13690_2023_1118_MOESM2_ESM.docx]

**Additional File 2**. Association between non-response and urban environment indicators. Models A are univariate regression models. Models B are adjusted regression models for gender, age, reported household income and year of the BHIS.

|  |  | **Models A**  **Univariate models** |  | **Models B**  **Adjusted models** |  |
| --- | --- | --- | --- | --- | --- |
|  |  | **OR (95% IC)** | **p value** | **OR (95% IC)** | **p value** |
| **Street canyon effect** | Tertile 1 (low) vs 3 (high) | 0.87 (0.71-1.06) | 0.177 | 0.8 (0.65-0.99) | 0.040 |
|  | Tertile 2 vs 3 (high) | 0.89 (0.72-1.09) | 0.266 | 0.9 (0.73-1.11) | 0.331 |
|  | No data vs Tertile 3 (high) | 0.65 (0.34-1.25) | 0.200 | 1.01 (0.49-2.11) | 0.973 |
| **Street corridor effect** | <median vs maximum | 0.87 (0.71-1.07) | 0.179 | 0.8 (0.65-0.99) | 0.044 |
|  | >=median vs maximum | 0.9 (0.74-1.11) | 0.324 | 0.86 (0.69-1.06) | 0.150 |
|  | No data vs maximum | 0.67 (0.35-1.28) | 0.223 | 1.03 (0.49-2.13) | 0.944 |
| **Linear treet density** | <median vs>=median | 0.97 (0.8-1.18) | 0.774 | 0.99 (0.81-1.21) | 0.940 |
|  | No tree vs>=median | 0.95 (0.76-1.18) | 0.629 | 0.98 (0.78-1.22) | 0.842 |
|  | No data vs>=median | 0.69 (0.36-1.33) | 0.269 | 1.11 (0.53-2.31) | 0.780 |
| **Vegetation coverage (1 km buffer)** | Tertile 1 (low) vs 3 (high) | 1.37 (1.11-1.69) | 0.003 | 1.44 (1.16-1.78) | 0.001 |
|  | Tertile 2 vs 3 (high) | 1.18 (0.96-1.46) | 0.109 | 1.25 (1-1.56) | 0.051 |
|  | No data vs Tertile 3 (high) | 0.89 (0.47-1.69) | 0.726 | 1.45 (0.7-2.98) | 0.314 |
| **Street visible vegetation coverage (within 10m)** | Tertile 1 (low) vs 3 (high) | 1.19 (0.97-1.46) | 0.095 | 1.29 (1.04-1.59) | 0.022 |
|  | Tertile 2 vs 3 (high) | 0.92 (0.75-1.13) | 0.413 | 1.01 (0.81-1.26) | 0.915 |
|  | No data vs Tertile 3 (high) | 0.73 (0.38-1.41) | 0.351 | 1.24 (0.59-2.58) | 0.574 |
| **View of green** | Tertile 1 vs 3 (high) | 1.09 (0.88-1.34) | 0.432 | 1.11 (0.89-1.38) | 0.348 |
|  | Tertile 2 vs 3 (high) | 0.9 (0.73-1.11) | 0.329 | 0.9 (0.72-1.12) | 0.355 |
|  | No data vs Tertile 3 (high) | 0.59 (0.41-0.85) | 0.004 | 0.6 (0.42-0.86) | 0.005 |
| **Noise from multiple sources (Lden)** | Tertile 1 (low) vs 3 (high) | 0.97 (0.8-1.19) | 0.785 | 0.91 (0.73-1.13) | 0.399 |
|  | Tertile 2 vs 3 (high) | 1.27 (1.03-1.56) | 0.024 | 1.21 (0.98-1.5) | 0.082 |
|  | No data vs Tertile 3 (high) | 0.77 (0.41-1.45) | 0.419 | 1.16 (0.57-2.34) | 0.685 |
| **Black carbon** | Tertile 1 (low) vs 3 (high) | 1.03 (0.85-1.27) | 0.741 | 0.71 (0.56-0.89) | 0.003 |
|  | Tertile 2 vs 3 (high) | 0.95 (0.77-1.16) | 0.591 | 0.8 (0.65-0.99) | 0.039 |
