# Supplementary material for: Non-response bias in the analysis of the association between mental health and the urban environment: a cross-sectional study in Brussels, Belgium
Source: Arch Public Health. 2023 Jul 7;81:129. doi: 10.1186/s13690-023-01118-y (PMC10327324; doi:10.1186/s13690-023-01118-y)
Supplement: Supplementary file 3 — Additional file 3. Association between non-response to depressive disorders related questions and socio-economic indicators (univariate regression models C) and between depressive disorders and socio-economic indicators (univariate regression models D). [file 13690_2023_1118_MOESM3_ESM.docx]

**Additional File 3.** Association between non-response to depressive disorders related questions and socio-economic indicators (univariate regression models C) and between depressive disorders and socio-economic indicators (univariate regression models D).

|  |  | **Models C (univariate)** |  | **Models D (univariate)** |  |
| --- | --- | --- | --- | --- | --- |
|  |  | **OR (95% IC)** | **p value** | **OR (95% IC)** | **p value** |
| **Reported household income** | Quartile 1 (low) vs 4 (high) | 2.47 (1.85-3.28) | 0.000 | 2.59 (1.71-3.93) | 0.000 |
|  | Quartile 2 vs 4 (high) | 2.19 (1.64-2.92) | 0.000 | 2.4 (1.57-3.68) | 0.000 |
|  | Quartile 3 vs 4 (high) | 1.36 (1.02-1.83) | 0.038 | 1.35 (0.88-2.05) | 0.168 |
|  | No answer vs Quartile 4 (high) | 3.83 (2.87-5.12) | 0.000 | 1.53 (0.91-2.58) | 0.108 |
| **Age** | 15–24vs 25–44 | 1.98 (1.52-2.56) | 0.000 | 0.51 (0.31-0.84) | 0.009 |
|  | 45–64vs 25–44 | 0.99 (0.82-1.19) | 0.901 | 1.17 (0.86-1.59) | 0.316 |
|  | 65+ vs 25–44 | 1.18 (0.96-1.45) | 0.106 | 1.23 (0.88-1.72) | 0.230 |
| **Gender** | M vs F | 1.07 (0.93-1.22) | 0.345 | 0.53 (0.41-0.69) | 0.000 |
| **Year of the BHIS** | 2013 vs 2008 | 2.26 (1.91-2.68) | 0.000 | 1.36 (1.05-1.77) | 0.021 |
| **Family composition** | Couple with child (ren) vs Single | 2.06 (1.65-2.56) | 0.000 | 0.46 (0.32-0.67) | 0.000 |
|  | Couple without child (ren) vs Single | 1.15 (0.9-1.48) | 0.262 | 0.61 (0.42-0.88) | 0.009 |
|  | One parent with child (ren) vs Single | 1.52 (1.15-2) | 0.003 | 1.13 (0.77-1.68) | 0.529 |
|  | Other/unknown vs Single | 2.52 (1.81-3.51) | 0.000 | 0.5 (0.3-0.83) | 0.007 |
| **Highest educational level in the household** | Higher secondary vs Higher | 1.34 (1.07-1.67) | 0.009 | 1.63 (1.18-2.25) | 0.003 |
|  | Lower secondary vs Higher | 1.78 (1.36-2.31) | 0.000 | 2.26 (1.53-3.35) | 0.000 |
|  | No diploma or primary education vs Higher | 3.57 (2.68-4.76) | 0.000 | 3.21 (2.05-5.02) | 0.000 |
|  | No answer vs Higher | 1.56 (1.01-2.42) | 0.046 | 1.32 (0.52-3.32) | 0.559 |
