# Supplementary material for: Non-response bias in the analysis of the association between mental health and the urban environment: a cross-sectional study in Brussels, Belgium
Source: Arch Public Health. 2023 Jul 7;81:129. doi: 10.1186/s13690-023-01118-y (PMC10327324; doi:10.1186/s13690-023-01118-y)
Supplement: Supplementary file 4 — Additional file 4. Association between non-response to depressive disorders related questions and socio-economic indicators (fully adjusted regression model E) and between depressive disorders and socio-economic indicators (fully adjusted regression model F). [file 13690_2023_1118_MOESM4_ESM.docx]

**Additional File 4.** Association between non-response to depressive disorders related questions and socio-economic indicators (fully adjusted regression model E) and between depressive disorders and socio-economic indicators (fully adjusted regression model F).

|  |  | **Model E (fully adjusted)** |  | **Model F (fully adjusted)** |  |
| --- | --- | --- | --- | --- | --- |
|  |  | **OR (95% IC)** | **p value** | **OR (95% IC)** | **p value** |
| **Reported household income** | Quartile 1 (low) vs 4 (high) | 2.2 (1.6-3.04) | 0,000 | 2.74 (1.65-4.54) | 0,000 |
|  | Quartile 2 vs 4 (high) | 2.12 (1.56-2.87) | 0,000 | 2.28 (1.42-3.67) | 0,001 |
|  | Quartile 3 vs 4 (high) | 1.37 (1.02-1.83) | 0,035 | 1.33 (0.85-2.09) | 0,209 |
|  | No answer vs Quartile 4 (high) | 4.15 (3.04-5.67) | 0,000 | 1.72 (0.99-2.97) | 0,053 |
| **Age** | 15–24vs 25–44 | 1.71 (1.28-2.29) | 0,000 | 0.46 (0.27-0.78) | 0,004 |
|  | 45–64vs 25–44 | 0.98 (0.81-1.2) | 0,871 | 1.15 (0.84-1.58) | 0,387 |
|  | 65+ vs 25–44 | 1.23 (0.96-1.58) | 0,106 | 0.88 (0.59-1.3) | 0,515 |
| **Gender** | M vs F | 1.07 (0.92-1.24) | 0,392 | 0.54 (0.42-0.7) | 0,000 |
| **Year of the BHIS** | 2013 vs 2008 | 2.96 (2.45-3.58) | 0,000 | 1.72 (1.3-2.29) | 0,000 |
| **Family composition** | Couple with child (ren) vs Single | 2.13 (1.65-2.74) | 0,000 | 0.47 (0.31-0.71) | 0,000 |
|  | Couple without child (ren) vs Single | 1.3 (1-1.69) | 0,055 | 0.71 (0.48-1.04) | 0,081 |
|  | One parent with child (ren) vs Single | 1.22 (0.89-1.68) | 0,215 | 0.84 (0.54-1.28) | 0,410 |
|  | Other/unknown vs Single | 2.41 (1.7-3.4) | 0,000 | 0.5 (0.29-0.85) | 0,011 |
| **Highest educational level in the household** | Higher secondary vs Higher | 1.22 (0.97-1.53) | 0,094 | 1.42 (1-2.01) | 0,049 |
|  | Lower secondary vs Higher | 1.62 (1.2-2.18) | 0,002 | 1.66 (1.09-2.53) | 0,018 |
|  | No diploma or primary education vs Higher | 3.26 (2.32-4.57) | 0,000 | 2.49 (1.51-4.11) | 0,000 |
|  | No answer vs Higher | 1.8 (1.11-2.92) | 0,018 | 0.96 (0.37-2.45) | 0,929 |
